# Revision of the genus *Cuvierina* Boas, 1886 based on integrative taxonomic data, including the description of a new species from the Pacific Ocean (Gastropoda, Thecosomata)

**DOI:** 10.3897/zookeys.619.10043

**Published:** 2016-09-27

**Authors:** Alice K. Burridge, Arie W. Janssen, Katja T.C.A. Peijnenburg

**Affiliations:** 1 Naturalis Biodiversity Center, P.O. Box 9517, 2300 RA Leiden, The Netherlands; 2 Institute for Biodiversity and Ecosystem Dynamics (IBED), University of Amsterdam, P.O. Box 94248, 1090 GE Amsterdam, The Netherlands

**Keywords:** Integrative taxonomy, DNA barcoding, geometric morphometrics, pteropods, biogeography

## Abstract

Shelled pteropods (Gastropoda, Thecosomata, Euthecosomata) are a group of holoplanktonic gastropods that occur predominantly in the surface layers of the world’s oceans. Accurate species identifications are essential for tracking changes in species assemblages of planktonic gastropods, because different species are expected to have different sensitivities to ocean changes. The genus *Cuvierina* has a worldwide warm water distribution pattern between ~36°N and ~39°S. Based on an integrative taxonomic approach combining morphometric, genetic, and biogeographic information, the two subgenera of *Cuvierina*, *Cuvierina* *s. str.* and *Urceolarica*, are rejected. A new species is introduced: *Cuvierina tsudai* **sp. n.**, which has to date been considered the same species as *Cuvierina pacifica*. *Cuvierina tsudai* **sp. n.** is endemic to the Pacific Ocean and is characterised by a shell height of 7.2-8.0 mm, a moderately cylindrical shell shape, the absence of micro-ornamentation and a triangular aperture. *Cuvierina pacifica* is restricted to the centre of the oligotrophic southern Pacific gyre, has a shell height of 6.6-8.5 mm, a more cylindrical shell shape, no micro-ornamentation and a less triangular aperture than *Cuvierina tsudai* **sp. n.**

## Introduction

Pteropods are holoplanktonic heterobranch gastropods classified in a superorder comprised of the orders Thecosomata and Gymnosomata, commonly referred to as “sea butterflies” and “sea angels”, respectively (Lalli and Gilmer 1989, Pierrot-Bults and Peijnenburg 2015). The order Thecosomata consists of Euthecosomata that have sinistrally coiled or straight, bilaterally symmetrical shells, and Pseudothecosomata that have either sinistrally coiled shells, an internal gelatinous pseudoconch, or are shell-less in the adult stage (Meisenheimer 1905, Tesch 1913). Pteropods play an important role in marine food webs (Jörger et al. 2010), and although most species occur in warm tropical and subtropical waters, the highest abundances have been observed for some (sub)polar cold water species (Bé and Gilmer 1977, Van der Spoel and Heyman 1983, Bednaršek et al. 2012, Burridge et al. 2016). Because of their thin-walled, aragonite shells, euthecosomes are exceptionally vulnerable to the effects of ocean acidification (e.g., Fabry et al. 2008, Bednaršek and Ohman 2015, Gattuso et al. 2015, Moya et al. 2016).

The genus *Cuvierina* is a remarkable group of shelled pteropods with relatively large (5.1-11.1 mm), straight, bottle-shaped shells (Janssen 2005). Ever since *Cuvierina* was described as a mollusc genus (as *Cuvieria* Rang, 1827, emended by Boas 1886), it has often been considered to consist of a single species, *Cuvierina columnella* (Rang, 1827), the type species of the genus by monotypy. The first taxonomic division within the genus came with the description of a second *Cuvierina* species, introduced as *Cuvieria urceolaris* (Mörch, 1850), but in later literature it was often interpreted as a form or subspecies of *Cuvierina columnella* (e.g., Tesch 1913, Van der Spoel 1967, Rampal 1975). A third form, Cuvierina columnella f. atlantica, was described by Van der Spoel (1970), and validated as a taxon of the species group by Bé et al. (1972). Bé and Gilmer (1977) interpreted the morphological differences between the three taxa as infraspecific variability. Contrarily, Rampal (2002) distinguished these taxa as independent species but introduced the taxon *Cuvierina spoeli* to replace the taxonomically invalid Cuvierina columnella f. atlantica. Because the holotype of *Cuvierina spoeli* was from the Indian Ocean, where *Cuvierina atlantica* is absent, it rather represented *Cuvierina columnella* and was rejected as a valid species by Janssen (2005). Two further extant species, *Cuvierina cancapae* and *Cuvierina pacifica*, were described by Janssen (2005).

According to the most recent taxonomic revision of *Cuvierina*, five extant species were assigned to two subgenera based on shell morphology and supposed lineages of fossil occurrences since the early Miocene (Janssen 2005, 2006). The subgenus Cuvierina *s. str.* consisted of *Cuvierina atlantica*, *Cuvierina columnella*, and *Cuvierina pacifica*, which are characterised by relatively slender, cylindrical shells, triangular rather than kidney-shaped apertures and the presence (*Cuvierina columnella*) or absence (*Cuvierina atlantica*, *Cuvierina pacifica*) of micro-ornamentation. Two geographical varieties were recognised within *Cuvierina pacifica*, one from the North Pacific and the other from the South Pacific, but were not formally introduced as new species. The subgenus Urceolarica, containing *Cuvierina cancapae* and *Cuvieria urceolaris*, is characterised by more inflated, bottle-shaped rather than cylindrical shells, pronounced micro-ornamentation, and kidney-shaped rather than triangular apertures.

All extant *Cuvierina* species are restricted to the surface layers of tropical and subtropical waters from ~45°N to ~40°S. In the Atlantic Ocean, *Cuvierina atlantica* occurs in the subtropical gyres and *Cuvierina cancapae* is found in tropical waters. In the Indian Ocean, *Cuvierina columnella* is found in the southern subtropical zone and *Cuvieria urceolaris* occurs in tropical waters and further south along Madagascar towards South Africa. *Cuvierina columnella* and *Cuvieria urceolaris* also occur in the Pacific Ocean along with *Cuvierina pacifica* (Janssen 2005, Burridge et al. 2015).

Burridge et al. (2015) examined the diversity, distribution, and evolution of *Cuvierina* taxa using integrative geometric morphometric, molecular, and biogeographic methods. They confirmed that the five species described for *Cuvierina* species have significantly different shell shapes and that *Cuvierina pacifica* consists of two disjunct morphometric groups, registered as *Cuvierina pacifica* N and *Cuvierina pacifica* S in their study. Three genetic lineages were distinguished based on mitochondrial Cytochrome Oxidase I DNA: the Atlantic lineage with *Cuvierina atlantica* and *Cuvierina cancapae*, the Indo-Pacific lineage with *Cuvierina columnella*, *Cuvieria urceolaris*, and *Cuvierina pacifica* N, and the South Pacific lineage with *Cuvierina pacifica* S. A new taxonomic description of *Cuvierina pacifica* N is required because the holotype of *Cuvierina pacifica* has the shell shape of *Cuvierina pacifica* S.

Based on the findings of Janssen (2005) and the integrative approach of Burridge et al. (2015) the taxonomy of the genus *Cuvierina* is revised. The subgenera *Cuvierina* *s. str.* and *Urceolarica* are rejected, a new species, *Cuvierina tsudai*, is described from the Pacific Ocean, and the species description of *Cuvierina pacifica* is restricted to the South Pacific lineage. A taxonomic key is provided for the identification of *Cuvierina* species.

## Methods

Two approaches were used to distinguish between *Cuvierina tsudai* and *Cuvierina pacifica* based on differences in shell shape. First, simple measurements of shell height and width, aperture diameters, and position of maximum shell width as applied to museum specimens by Janssen (2005) were used to distinguish between *Cuvierina tsudai* and *Cuvierina pacifica*. Second, geometric morphometric data of shell shapes in ventral and apertural orientations were used for 168 adult specimens of *Cuvierina* that were registered as *Cuvierina pacifica* N or *Cuvierina pacifica* S in Burridge et al. (2015). The specimens corresponded to museum specimens as identified and measured by Janssen (2005, N = 92), additional museum specimens (N = 24), and recently collected fresh specimens (N = 52). Geometric morphometric methods consisted of digitising shell outlines using tpsDig and tpsUtil (Rohlf 2006) to contain 76 ventral and 37 apertural semi-landmarks per shell, after which a generalised least square Procrustes superimposition was applied (GLS, Kendall 1977 in Zelditch et al. 2004) to rotate, translate, and scale the semi-landmark coordinates. A subsequent thin-plate spline (TPS) analysis (e.g., Zelditch et al. 2004) provided centroid sizes, a size measure depending on surface area, and multiple relative warp axes per specimen, containing information on shape. To describe the new species *Cuvierina tsudai* as well as to reject the validity of the *Cuvierina* subgenera, Cytochrome Oxidase I mitochondrial (COI) DNA and 28S ribosomal DNA sequence data from Burridge et al. (2015) were used.

## Results and discussion

### Distinction between *Cuvierina tsudai* and *Cuvierina pacifica*

**Figure 1.**
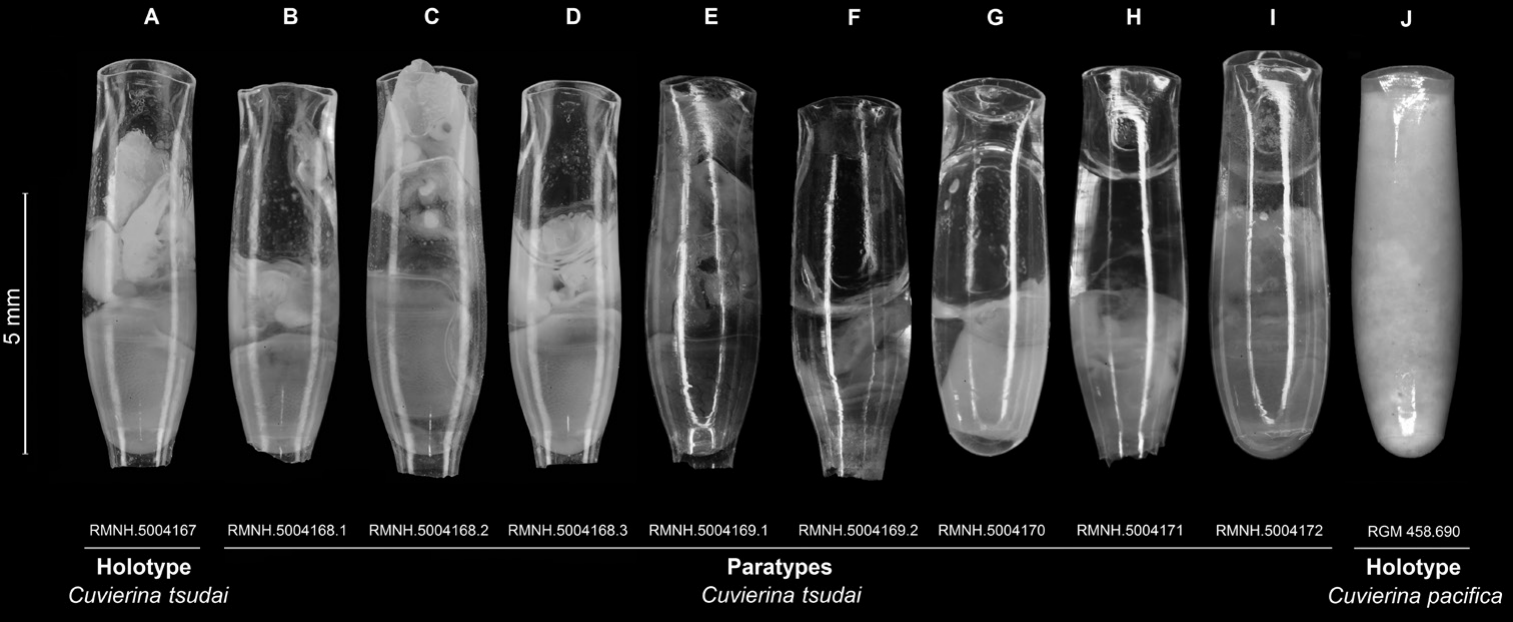
Holotype and paratypes of *Cuvierina tsudai* and holotype of *Cuvierina pacifica*. **A** Holotype (RMNH.5004167) and **B–I** paratypes (RMNH.5004168-72) of *Cuvierina tsudai* and **J** holotype of *Cuvierina pacifica* (RGM 458.690) photographed in a ventral view. Photographs of RMNH.5004169-72 from Burridge et al. (2015); RMNH.5004167-68 taken by R. van der Hulst and RGM 458.692 taken by E.F. de Vogel, this study. RMNH = Naturalis Biodiversity Center, mollusc collection and RGM = Naturalis Biodiversity Center, fossil planktonic mollusc collection, Leiden.

**Figure 2.**
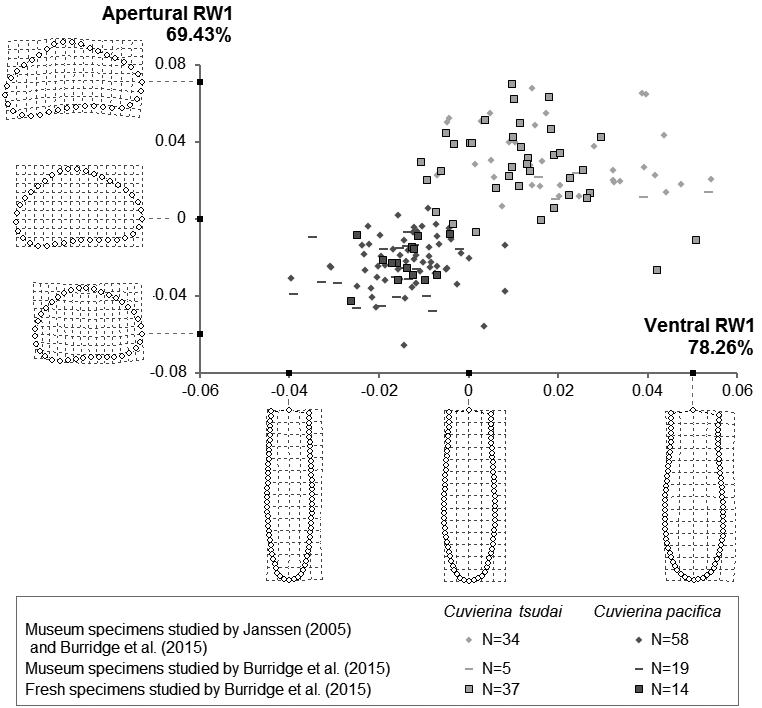
Shape variation in *Cuvierina tsudai* and *Cuvierina pacifica* by means of Relative Warp (RW) data. Ordination of RW data of *Cuvierina tsudai* and *Cuvierina pacifica* for the first ventral and apertural RWs (N = 167 excluding 1 specimen with only one orientation). On the X-axis, RW1 depicts 78.26% of the total ventral shape variation. On the Y-axis, 69.43% of the apertural shape variation is explained by its RW1. Shape variations depicted by ventral and apertural RW1 (with subsequent RWs = 0) are shown.

*Cuvierina tsudai* and *Cuvierina pacifica* are similar in size but have different shell shapes, COI mtDNA and 28S rDNA. Because of their Pacific distributions and similarities in shell size, *Cuvierina tsudai* and *Cuvierina pacifica* have to date been considered the same species. Although Janssen (2005) demonstrated their presence as morphological varieties within *Cuvierina pacifica*, the congruence between morphometric and genetic differentiation supports the separation into two species (Figs 1A–J and 2, fig. 4 in Burridge et al. 2015). Shell heights of *Cuvierina tsudai* specimens are between 7.2 and 8.8 mm, showing a large overlap with *Cuvierina pacifica*, which measures between 6.6 and 8.5 mm (Janssen 2005). However, in terms of shell shape, *Cuvierina pacifica* and *Cuvierina tsudai* are significantly different (fig. 29 lower left in Janssen 2005, Burridge et al. 2015). The shell of *Cuvierina tsudai* is wider (more inflated) than the slender and more cylindrical *Cuvierina pacifica* (Fig. 2). *Cuvierina pacifica* has a larger height/width-ratio between 3.25 and 3.96 (mean 3.50) compared to *Cuvierina tsudai*, which has a ratio between 2.77 and 3.46 (mean 3.14). The position of maximum shell width is located at 34-45% (mean 40%) of the shell height from the septum upwards for *Cuvierina pacifica* and at 33-42% (mean 37%) for *Cuvierina tsudai* (Janssen 2005). The aperture of *Cuvierina tsudai* is wider, more triangular and more concave on the ventral side than in *Cuvierina pacifica*. The overall shape variation is larger for *Cuvierina tsudai* than for *Cuvierina pacifica* (Fig. 2). The average pairwise genetic distance of COI mtDNA (658bp fragment) between *Cuvierina tsudai* (N = 16) and *Cuvierina pacifica* (N = 43) is 4.5%. The genetic variation of COI within *Cuvierina tsudai* is 1.6% compared to 0.8% within *Cuvierina pacifica*. The 28S rDNA fragment (965bp) of *Cuvierina tsudai* differs at least at one position compared with other *Cuvierina* species, except for *Cuvierina columnella* (Burridge et al. 2015).

The larger genetic and shell shape variation for *Cuvierina tsudai* compared to *Cuvierina pacifica* coincides with a much larger Pacific distribution and lower ecological specificity of *Cuvierina tsudai*. *Cuvierina pacifica* is restricted to the centre of the oligotrophic southern Pacific gyre and occupies a more specialised ecological niche based on ecological niche modelling (ENM) than *Cuvierina tsudai* (Burridge et al. 2015). This study used presence-only data and six uncorrelated environmental parameters, of which ocean surface temperature and chlorophyll *a* concentration were the most important. The distribution of *Cuvierina tsudai* was mostly explained by maximum monthly sea surface temperatures (SST) and near-surface chlorophyll *a* concentrations (both 30.8%). The distribution of *Cuvierina pacifica* was best explained by low maximum monthly chlorophyll *a* concentrations (57.1%).

### Description of *Cuvierina tsudai* sp. n.

**Superfamily Cavolinioidea Gray, 1840**

**Family Cuvierinidae Gray, 1840**

**Genus *Cuvierina* Boas, 1886 (= replacement name for *Cuvieria* Rang, 1827 non Lesueur & Petit, 1807, pl. 30 (Coelenterata)**

**Type species.** *Cuvieria columnella* Rang, 1827, p. 323, pl. 45 figs 1–3, by monotypy.

#### Cuvierina tsudai sp. n.

Taxon classificationAnimaliaThecosomataCuvierinidae

http://zoobank.org/B33A28E9-BCDE-4F2B-9349-F3E18CCD87BE

Cuvieria columnella Rang, 1827: 323 (partim).
Cuvierina columnella : Boas 1886: 132, 217, pl. 6 fig. 95g (*partim*, *non* Rang); Rampal 2002: 214 (*partim*, *non* Rang).
Cuvierina columnella (Rang, 1827) forma columnella (Rang, 1827) – Van der Spoel 1967: 79 (*partim, non* Rang);Van der Spoel 1970: 120, fig. 19 (*partim*, *non* Rang).
Cuvierina (Cuvierina) pacifica Janssen, 2005: 46 figs. 18-20 (*partim*, northern Pacific specimens only, *non* figs. 14-17 = Cuvierina pacifica).
Cuvierina pacifica N (Janssen, 2005): Burridge et al. 2015: 5, fig. 2.

##### Holotype.

RMNH.5004167, also see Fig. 1A and Table 1.

**Table 1.** Voucher and sampling information of type specimens of *Cuvierina tsudai* including the holotype of *Cuvierina pacifica*.

Museum voucher	Image voucher	Collection date	Latitude	Longitude	Cruise	Station	COI GenBank	28S GenBank	First studied
Holotype of *Cuvierina tsudai*									
RMNH.5004167	C_PNE_SE1201_21_01	2012-05-15	8°47'N	158°49'W	SE1201	21			This study
Paratypes of *Cuvierina tsudai*									
RMNH.5004168.1	C_PNE_SE1201_21_02	2012-05-15	8°47'N	158°49'W	SE1201	21			This study
RMNH.5004168.2	C_PNE_SE1201_21_03	2012-05-15	8°47'N	158°49'W	SE1201	21			This study
RMNH.5004168.3	C_PNE_SE1201_21_04	2012-05-15	8°47'N	158°49'W	SE1201	21			This study
RMNH.5004169.1	C_PNE_KH1110_08_01	2011-12-19	22°47'N	158°06'W	KH-11-10	8	KP292730	KP292636	Burridge et al. 2015
RMNH.5004169.2	C_PNE_KH1110_08_20	2011-12-19	22°47'N	158°06'W	KH-11-10	8	KP292748	KP292637	Burridge et al. 2015
RMNH.5004170	C_PNE_KM1109_02_02	2011-03-04	21°14'N	158°11'W	Kilo Moana 1109	2	KP292755	KP292639	Burridge et al. 2015
RMNH.5004171	C_PNE_KM1109_08_01	2011-03-06	21°20'N	158°22'W	Kilo Moana 1109	8	KP292759	KP292640	Burridge et al. 2015
RMNH.5004172	C_PNW_TMKT1020_05_01	2010-09-29	27°08'N	125°33'E	R/V Tansei-Maru KT-10-20	5	KP292766	KP292642	Burridge et al. 2015
ZMUC, not registered	Figure 18	1933-08-21	33°45'N	137°30'W	DANA	4794			Janssen 2005
ZMUC, not registered	Figure 19	1934-02-12	32°56'N	131°50'W	DANA	4807			Janssen 2005
ZMUC, not registered	Figure 20	1929-05-25	20°04'N	125°59'E	DANA	3718 V			Janssen 2005
Holotype of *Cuvierina pacifica*									
RGM 458.692	Figure 15	1986-04/05	18°39'S	172°12'W	Manihiki Plateau Expedition	U351a			Janssen 2005

##### Type locality.

8°47'N, 158°49'W.

##### Paratypes.

See Fig. 1B–I and Table 1 for all specimen information. Three specimens from the type locality (RMNH.5004168); three specimens from the Zoological Museum of the University of Copenhagen, Denmark (ZMUC, not registered) illustrated by Janssen (2005, figs. 18–20); five specimens from four locations (RMNH.5004169–72) studied by Burridge et al. (2015, referred to as *Cuvierina pacifica* N therein). The latter five specimens have COI mtDNA and 28S rDNA sequences available at GenBank (see Table 1).

##### Additional material examined.

Specimens recorded as *Cuvierina pacifica* from the North Pacific Ocean in Janssen (2005: 49, 71), housed in the Muséum National d’Histoire Naturelle (MNHN, Paris, France) and ZMUC (Copenhagen, Denmark). Specimens from Burridge et al. (2015), referred to as *Cuvierina pacifica* N in Table S1 therein, with photographs deposited at the Dryad repository (https://doi.org/10.5061/dryad.7n1q4) and COI mtDNA (KP292730-72) and 28S rDNA sequences (KP292636-42) deposited at GenBank. These specimens are housed in Naturalis Biodiversity Center (Leiden, The Netherlands) and ZMUC (Copenhagen, Denmark). Registration numbers, if available, from Janssen (2005).

##### Diagnosis.

Shell moderately small, adult specimens 7.2–8.8 mm high, height/width-ratio 2.77–3.46 (mean 3.14), position of maximum shell width 33–42% (mean 37%) of shell height from septum upwards. Aperture triangular. No longitudinal micro-ornamentation.

##### Description.

**Figure 3.**
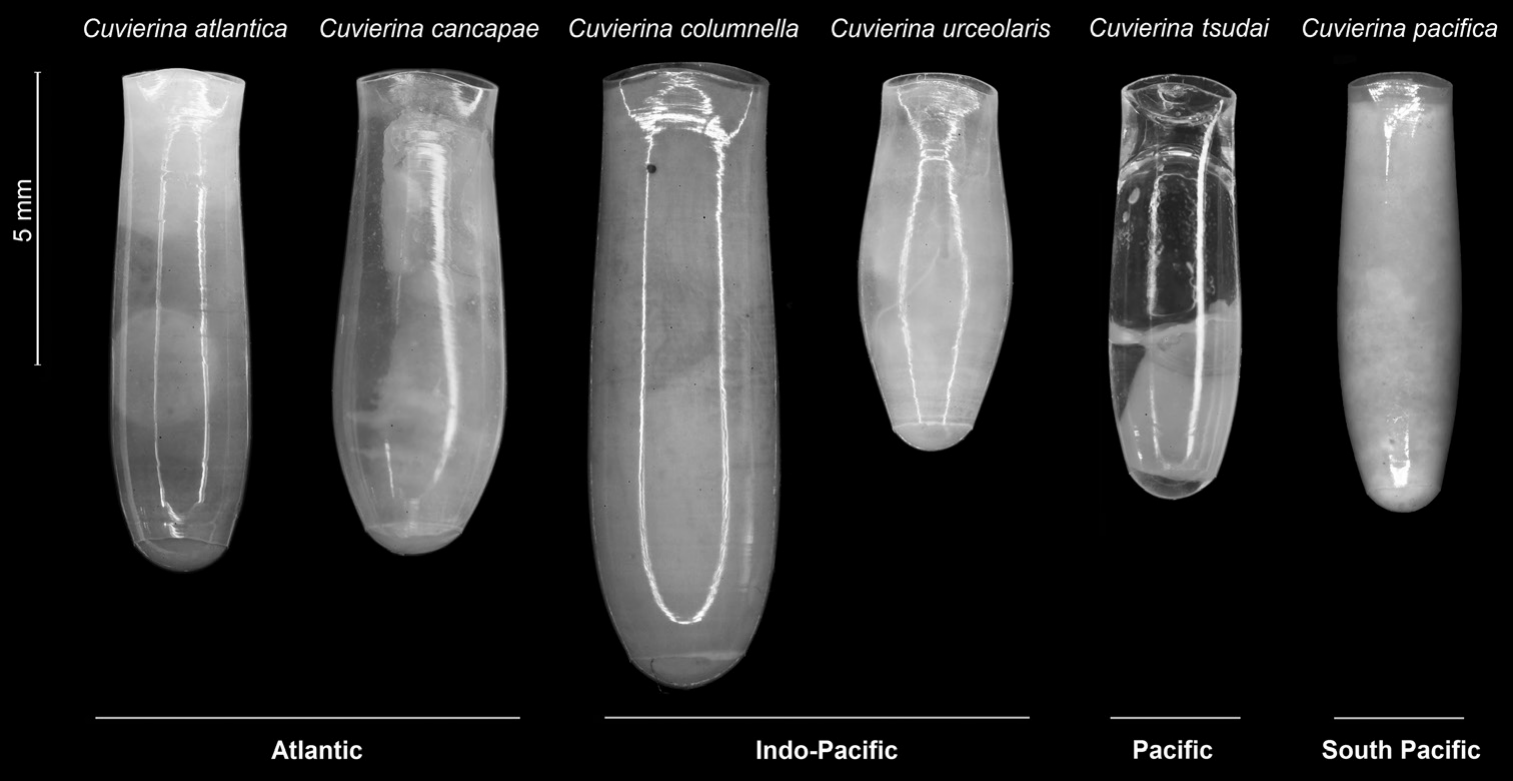
Typical specimens of six *Cuvierina* species.

The shell shape of *Cuvierina tsudai* differs from other *Cuvierina* species. Its shell height is smaller than in *Cuvierina columnella*, *Cuvierina cancapae*, and *Cuvierina atlantica*, but larger than in *Cuvieria urceolaris*, and of similar size compared to *Cuvierina pacifica*. The position of maximum shell width is distinctly higher than for *Cuvierina columnella* and *Cuvierina atlantica* and lower than for *Cuvierina pacifica*. It is more cylindrical in shape than the inflated (bottle-shaped) *Cuvieria urceolaris* but less cylindrical than *Cuvierina atlantica* and *Cuvierina pacifica*. It differs from *Cuvieria urceolaris* and *Cuvierina cancapae* by the absence of micro-ornamentation. It has a more triangular and wider aperture than *Cuvieria urceolaris* and *Cuvierina pacifica* (Fig. 3, Janssen 2005, Burridge et al. 2015).

##### Distribution.

*Cuvierina tsudai* has a wide, exclusively Pacific distribution between 36°N and 39°S, in which it co-exists with *Cuvierina columnella*, *Cuvieria urceolaris*, and *Cuvierina pacifica*. It has been found most often in the North Pacific, but also occurs in the South Pacific. It has not been found thus far in the central, oligotrophic parts of the South Pacific subtropical gyre, the southeast Pacific, the coral triangle west of the Philippines or southwest of Papua New Guinea.

##### Etymology.

Named after Atsushi Tsuda, professor in biological oceanography at the University of Tokyo, Japan, for sending us pteropod samples from the Pacific Ocean and in recognition of his services to the zooplankton research community.

### Rejection of the subgenera in *Cuvierina*

Two subgenera of *Cuvierina* were described that supposedly evolved since the early Miocene (Aquitanian, 23 million years ago): *Cuvierina* *s. str.*, with extant species *Cuvierina atlantica*, *Cuvierina columnella*, and *Cuvierina pacifica*, and *Urceolarica* with extant species *Cuvierina cancapae* and *Cuvieria urceolaris* (see Janssen 2005, 2006). They were based on distinguishing shell characteristics in fossil species such as the position of maximum shell width, aperture shape and presence or absence of micro-ornamentation. However, the morphology and molecular phylogenetic information of recent species are in conflict with this separation. *Cuvierina columnella*, typically a *Cuvierina* *s. str.* species, has distinct micro-ornamentation, which was considered one of the distinguishing characters of the subgenus Urceolarica. It was shown that there are three divergent and well-supported lineages based on genetic data: the Atlantic (*Cuvierina atlantica* and *Cuvierina cancapae*), Indo-Pacific (*Cuvierina columnella*, *Cuvieria urceolaris* and *Cuvierina tsudai*), and South Pacific (*Cuvierina pacifica*) lineages (fig. 4 in Burridge et al. 2015). Hence, we reject the two subgenera within *Cuvierina*.

### Taxonomic key to *Cuvierina* pteropods

The following taxonomic key identifies adult *Cuvierina* pteropod species based on distinctive shell shape characteristics and shell sizes. Photographs of typical adult shells are shown in Fig. 3.

**Table:** 

1	Micro-ornamentation present	**2**
–	Micro-ornamentation absent	**4**
2	Strongly inflated shell shape, shell height 5.1–6.7 mm	***Cuvieria urceolaris***
–	Moderately inflated or cylindrical shell shape, shell height 7.5–11.1 mm	**3**
3	Cylindrical shell shape, shell height 8.8–11.1 mm	***Cuvierina columnella***
–	Moderately inflated shell shape, shell height 7.5–9.3 mm	***Cuvierina cancapae***
4	Cylindrical shell shape and triangular aperture, shell height 6.7–10.5 mm	***Cuvierina atlantica***
–	Moderately inflated or cylindrical shell shape, triangular to kidney-shaped aperture, shell height 6.6–8.8 mm	**5**
5	Cylindrical shell shape and kidney-shaped aperture, shell height 6.6–8.5 mm	***Cuvierina pacifica***
–	Moderately inflated shell shape and triangular aperture, shell height 7.2–8.8 mm	***Cuvierina tsudai***

## Conclusions

Morphometric, genetic, and biogeographic information has led to the introduction of a new species of the warm water pteropod genus *Cuvierina* and the rejection of its subgenera. We encourage a combined evidence approach of taxonomy to more accurately identify species boundaries and higher taxonomic relationships in planktonic gastropods. Accurate taxonomic identification is a prerequisite to assess to what extent species are affected by ocean changes and to potentially use them as bioindicators.

## Supplementary Material

XML Treatment for Cuvierina tsudai
